# Improved Synthesis of Dinuclear [M(μ‐X)(η^5^‐Cp*)X]_2_ Precursors for Half‐Sandwich Complexes (M=Rh or Ir; X=Br or I)

**DOI:** 10.1002/open.202500026

**Published:** 2025-03-03

**Authors:** Kamila Petrželová, Ondřej Bárta, Renata Héžová, Alžběta Andrýsková, Jan Hošek, Pavel Štarha

**Affiliations:** ^1^ Department of Inorganic Chemistry Palacký University Olomouc 17. listopadu 1192/12 77146 Olomouc Czech Republic; ^2^ Department of Pharmacology and Toxicology Veterinary Research Institute Hudcova 296/70 62100 Brno Czech Republic; ^3^ Department of Experimental Physics Palacký University Olomouc 17. listopadu 1192/12 77146 Olomouc Czech Republic

**Keywords:** Rhodium, Iridium, Cyclopentadienyl, Bromido, Iodido

## Abstract

The long‐known dinuclear complexes of the general formula [M(μ‐X)(η^5^‐Cp*)X]_2_ (**1**–**4**; M=Rh or Ir, X=Br or I; HCp*=pentamethylcyclopentadiene) represent important intermediates for the preparation of biologically (*e. g*., anticancer or antimicrobial) and catalytically active half‐sandwich rhodium(III) and iridium(III) complexes. Here we report the optimized rapid microwave‐assisted syntheses of the mentioned dinuclear synthons **1**–**4**. With respect to their following synthetic utilization, we examined their antiproliferative activity in the representative human cancer cell lines, where they did not show any relevant cytotoxicity. However, the iridium complexes **3** and **4** exhibited considerable catalytic activity in a model transfer hydrogenation of acetophenone using propan‐2‐ol as the hydrogen source. The obtained comprehensive data sets can be useful in the evaluation of the ligand effects in complexes prepared from **1**–**4** and used in *e. g*. bioinorganic and catalytic applications.

## Graphical abstract

Dinuclear rhodium(III) and iridium(III) bromido and iodido cyclopentadienyl compounds serve as precursors of compounds for various applications (*e. g*., bioactive compounds or catalysts). This research focused on optimizing their synthesis through a rapid microwave‐assisted method, achieving completion within 5 min. Dimers demonstrated no toxicity in selected lung cancer cells, while Ir compounds exhibited moderate catalytic activity in the hydrogenation of acetophenone.

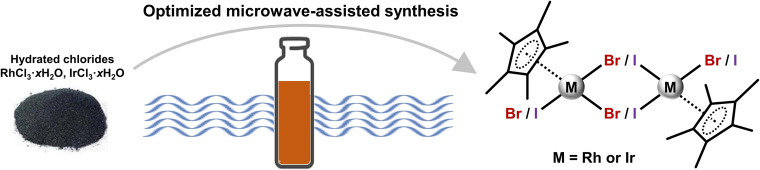

## Introduction

In the field of anticancer and anti‐microbial metal‐based compounds,^[1]^ half‐sandwich complexes of iridium and rhodium represent pharmacologically prospective and progressive structural types with numerous highly effective examples.[^2^, ^3^, ^4^] The main advantage besides their high biological effect is their selectivity towards the cancer cells over the normal ones and their mechanism of action, which seems to be different from conventional anticancer platinum‐based drugs (*e. g*., cisplatin).

The first representatives of anticancer Rh(III) and Ir(III) cyclopentadienyl complexes were reported in 2006 by Peruzzini, Dyson and co‐workers,^[5]^ and in 2007 by Sheldrick and co‐workers,^[6]^ respectively. These chlorido compounds were synthesized from dinuclear intermediates [Rh(μ‐Cl)(η^5^‐Cp*)Cl]_2_, and [Ir(μ‐Cl)(η^5^‐Cp*)Cl]_2_,^[7]^ respectively, which are the typical synthetic pathways in the field; HCp*=pentamethylcyclopentadiene. These dimers were earlier reported by Maitlis and co‐workers[^8^, ^9^] and their conventional synthesis (48 h, reflux, N_2_ atmosphere)^[10]^ was later optimized to shorter reaction time (<5 min) by using a microwave reactor without the need for inert atmosphere.[^11^, ^12^] Rh(III) and Ir(III) analogues involving different cyclopentadienyl derivatives can be acquired by the same conventional^[13]^ or microwave‐assisted[^12^, ^14^] methods.

**Figure 1:**
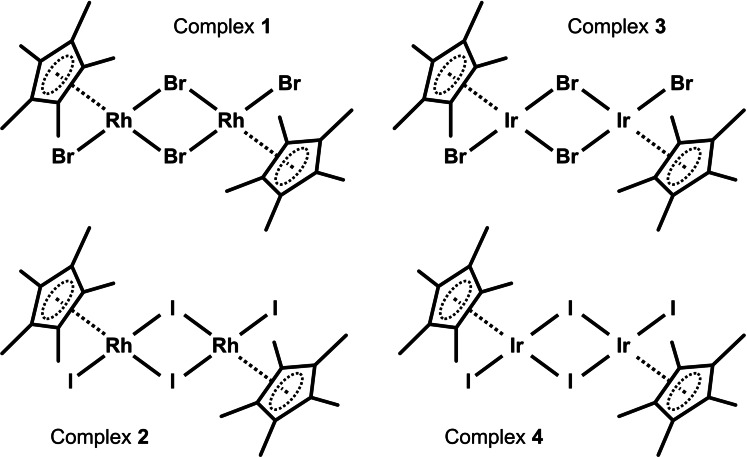
Structural formulas of the studied Rh(III) and Ir(III) complexes **1**–**4**.

Half‐sandwich Rh(III) and Ir(III) chlorido complexes have been widely studied for their biological activity.[^2^, ^3^, ^4^] In some works, the authors have also paid attention to Rh(III) and Ir(III) bromido and iodido complexes,[^15^, ^16^] which can be prepared from dimers [Rh(μ‐Br)(η^5^‐Cp*)Br]_2_, [Ir(μ‐Br)(η^5^‐Cp*)Br]_2_, [Ir(μ‐I)(η^5^‐Cp*)I]_2_ and [Rh(μ‐I)(η^5^‐Cp*)I]_2_ (Figure 1).[^9^, ^17^] In some cases, these bromido and iodido dimers were prepared *in situ* within the synthesis of the resulting complexes.^[18]^ Alternatively, the chlorido ligand can be subtracted by a silver salt (*e. g*., nitrate or triflate) from a respective monuclear chlorido complex and replaced with bromide or iodide using *e. g*., KBr or KI.^[19]^

In this work, we report on the optimized synthesis of dinuclear complexes [Rh(μ‐Br)(η^5^‐Cp*)Br]_2_ (**1**), [Rh(μ‐I)(η^5^‐Cp*)I]_2_ (**2**), [Ir(μ‐Br)(η^5^‐Cp*)Br]_2_ (**3**) and [Ir(μ‐I)(η^5^‐Cp*)I]_2_ (**4**) (Figure 1), whose syntheses, despite being known for several decades,[^9^, ^17^] can still be further improved.^[20]^ Complexes **1**–**4** were prepared in high yields in a microwave reactor (*t* <5 min) and characterized by relevant analytical techniques (NMR, mass spectrometry, FT‐IR, elemental analysis). Since these complexes represent important intermediates for the preparation of biologically and catalytically active complexes, we also studied their biological (antiproliferative activity in selected human cancer cell lines) and catalytic (transfer hydrogenation of acetophenone) activities, to obtain a comprehensive data set useful for comparison of these “background” activities of **1**–**4** with activities of products obtained from these synthetic precursors in the future.

## Experimental Section

### Materials

Chemicals (RhCl_3_⋅*x*H_2_O, IrCl_3_⋅*x*H_2_O, 1,2,3,4,5‐pentamethylcyclopenta‐1,3‐diene, potassium bromide (KBr), potassium iodide (KI), potassium hydroxide (KOH), 1,3,5‐trimethoxybenzene), solvents (methanol (MeOH), diethyl ether (DEE), propan‐2‐ol) and deuterated solvents (CDCl_3_, DMSO‐*d_6_*) were purchased from Merck (Prague, Czech Republic), VWR International (Stříbrná Skalice, Czech Republic), Litolab (Chudobín, Czech Republic) and Chemstar (Plzeň, Czech Republic), and they were used as received. The syntheses of the [Rh(μ‐Cl)(η^5^‐Cp*)Cl]_2_ and [Ir(μ‐Cl)(η^5^‐Cp*)Cl]_2_ complexes were conducted according to a previously published procedure.^[11]^

All cell culture media and supplements [foetal bovine serum (FBS), antibiotics] were purchased from Biosera (Cholet, France). A Cell Counting Kit 8 (CCK‐8) was obtained from Abcam (Cambridge, UK).

### General Methods

Syntheses were performed using the microwave reactor Monowave 300 (Anton Paar). Elemental analysis was performed by a Flash 2000 CHNS Elemental Analyser (Thermo Scientific). Electrospray ionization mass spectrometry (ESI‐MS; MeOH solutions) was carried out with an LCQ Fleet ion trap spectrometer (Thermo Scientific; QualBrowser software, version 2.0.7) in the positive ionization mode (ESI+; RI=Relative Intensity (%)).

^1^H and ^13^C NMR spectra of complexes **1**–**4** were recorded using DMSO‐*d_6_* solutions at 298 K on a Varian‐400 spectrometer (^1^H, 400 MHz; ^13^C, 101 MHz). The spectra were calibrated against the residual signals of the used solvent (^1^H at 2.50 ppm and ^13^C at 39.5 ppm). Proton resonances in the reported ^1^H spectra are defined as s=singlet. The NMR analyses of catalytic experiments were performed on JEOL ECZ400r spectrometer (^1^H, 400 MHz) and the recorded spectra referenced against tetramethylsilane as an internal standard.

Energy‐dispersive X‐ray spectroscopy (EDX) analysis was performed using a silicon detector XFlash 410 (Bruker Nano GmbH, Berlin, Germany) attached to a VEGA3 LMU scanning electron microscope (Tescan, Brno, Czech Republic). The accelerating voltage was set to 30 kV and the detector take‐off angle was 35°. EDX elemental analysis was performed using QUANTAX Esprit software.

A Jasco FT/IR‐4700 spectrometer was used for the collection of the infrared (IR) spectra of complexes in the range of 400–4000 cm^−1^ by using the attenuated total reflection (ATR) technique on a diamond plate. The spectral intensity is defined as w=weak, m=middle, s=strong and vs=very strong peak.

### Synthesis

For [Rh(μ‐X)(η^5^‐Cp*)X]_2_ (X=Br for **1**, and I for **2**), [Rh(μ‐Cl)(η^5^‐Cp*)Cl]_2_ (50 mg, 0.08 mmol) was suspended in MeOH (4 mL) in a microwave vial. 2 mL of a saturated aqueous solution of KBr or 1 mL of a saturated aqueous solution of KI was added for **1** and **2**, respectively. The mixtures were heated in the microwave reactor using a temperature program consisting of a 2 min ramp to 140 °C, 15 min (for **1**) or 30 min (for **2**) isothermal hold (140 °C), and cooling to 45 °C. The resulting brown‐red (for **1**) or dark purple (for **2**) suspensions were filtered and washed three times with 3 mL of MeOH, eight times with 5 mL water, and dried in a desiccator under reduced pressure.

The syntheses of Ir(III) complexes [Ir(μ‐X)(η^5^‐Cp*)X]_2_ (X=Br for **3**, and I for **4**) were carried out following the same procedures employed for Rh(III) compounds **1** and **2**, with [Ir(μ‐Cl)(η^5^‐Cp*)Cl]_2_ (50 mg, 0.06 mmol) utilized as the starting material.

[Rh(μ‐Br)(η^5^‐Cp*)Br]_2_ (**1**): Yield: 94 %. CHN calc. (found) for C_20_H_30_Br_4_Rh_2_: C, 30.18 (30.13); H, 3.80 (3.78) %. Mass spec. ESI+ (CH_3_OH, m/z): calc. (found) 714.8 (714.7) for [Rh_2_Br_3_(Cp*)_2_]^+^ (RI=60 %), 373.1 (373.1) for [Rh(Cp*)_2_]^+^ (RI=100 %), 316.9 (316.9) for [RhBr(Cp*)]^+^ (RI=35 %). ^1^H NMR (DMSO‐*d_6_*, 298 K): δ 1.73 (s, −C*H*_3_) ppm. ^13^C NMR (DMSO‐*d_6_*, 298 K): δ 99.6, 9.2 ppm. FTIR (υ, ATR, cm^−1^): 2958w, 2903w, 1443 s, 1367vs, 1159 m, 1074 m, 1013vs, 797w, 614 m, 581 m, 534 m.

[Rh(μ‐I)(η^5^‐Cp*)I]_2_ (**2**): Yield: 95 %. CHN calc. (found) for C_20_H_30_I_4_Rh_2_: C, 24.41 (24.80); H, 3.07 (3.01) %. Mass spec. ESI+ (CH_3_OH, m/z): calc. (found) 856.8 (856.6) for [Rh_2_I_3_(Cp*)_2_]^+^ (RI=100 %), 364.9 (364.9) for [RhI(Cp*)]^+^ (RI=75 %). ^1^H NMR (DMSO‐*d_6_*, 298 K): δ 1.95 (s, −C*H*_3_) ppm. ^13^C NMR (DMSO‐*d_6_*, 298 K): δ 100.8, 10.5 ppm. FTIR (υ, ATR, cm^−1^): 2957w, 2900s, 1455 s, 1367vs, 1153 m, 1074 m, 1009vs, 950w, 794w, 610 m, 532 m.

[Ir(μ‐Br)(η^5^‐Cp*)Br]_2_ (**3**): Yield: 88 %. CHN calc. (found) for C_20_H_30_Br_4_Ir_2_: C, 24.65 (24.74); H, 3.10 (3.04) %. Mass spec. ESI+ (CH_3_OH, m/z): calc. (found) 894.9 (894.7) for [Ir_2_Br_3_(Cp*)_2_]^+^ (RI=20 %), 407.0 (407.0) for [IrBr(Cp*)]^+^ (RI=100 %). ^1^H NMR (DMSO‐*d_6_*, 298 K): δ 1.71 (s, −C*H*_3_) ppm. ^13^C NMR (DMSO‐*d_6_*, 298 K): δ 93.0, 8.7 ppm. FTIR (υ, ATR, cm^−1^): 2984w, 2960w, 2905 m, 1448vs, 1371vs, 1159 m, 1079 m, 1029vs, 611 m, 581 m, 531 m.

[Ir(μ‐I)(η^5^‐Cp*)I]_2_ (**4**): Yield: 90 %. CHN calc. (found) for C_20_H_30_I_4_Ir_2_: C, 20.66 (20.63); H, 2.60 (2.45) %. Mass spec. ESI+ (CH_3_OH, m/z): calc. (found) 1034.9 (1034.7) for [Ir_2_I_3_(Cp*)_2_]^+^ (RI=5 %), 455.0 (455.1) for [IrI(Cp*)]^+^ (RI=10 %). ^1^H NMR (DMSO‐*d_6_*, 298 K): δ 1.88 (s, −C*H*_3_) ppm. ^13^C NMR (DMSO‐*d_6_*, 298 K): δ 94.4, 9.9 ppm. FTIR (υ, ATR, cm^−1^): 2957w, 2901 s, 1476vs, 1371 s, 1156 m, 1078 m, 1019vs, 950 m, 794w, 607 m, 532 m.

### Cell Culture

The cytotoxic effects of the tested complexes on the human lung cancer cell lines MOR (catalogue no. 84112312), A549 (catalogue no. 86012804), and on cisplatin‐resistant MOR/CPR (catalogue no. 96042333) were evaluated (all obtained from ECACC, Salisbury, UK). RPMI‐1640 culture medium was used for the MOR and MOR/CPR cell lines, and high‐glucose DMEM was used for the A549 cell line. Media were supplemented with 10 % FBS, antibiotics (100 U/mL penicillin and 100 mg/mL streptomycin), antimicrobial agent Normocin™ (100 μg/mL; Invivogen, San Diego, CA, USA) and 1 μg/mL cisplatin (CDDP) for the MOR/CPR cell line. The cells were passaged twice weekly. All experiments were performed on the 3^rd^–15^th^ passages after defrosting of cells when cells possess the highest stability and growth potential.

### In vitro Cytotoxicity Testing

Adherent A549, MOR, and MOR/CPR cells were seeded at a density of 1×10^4^ cells/well in 96‐well plates overnight. The next day, fresh complete cultivation media was added to the cells (without CDDP in the case of MOR/CPR cells), and the tested complexes were dissolved in DMF (the concentration of DMF never exceeded 0.1 % v/v in the presence of cells). Test compounds were evaluated at concentrations ranging from 1.25 to 160 μM. Cell viability was measured with a CCK8 according to the manufacturer's manual after 72 h of incubation. The relative cell viability was calculated as a ratio between cells treated with compounds and cells treated only with DMF (negative control).

### Catalytic Transfer Hydrogenation of Acetophenone

All glassware was treated with aqua regia and dried at 120 °C before use. Acetophenone (approximately 1.0 mmol) was weighed into a dry Schlenk flask. Corresponding amounts of 1,3,5‐trimethoxybenzene (1/3 mol. equiv. of acetophenone) and catalyst (1 mol. % to acetophenone, based on metal content), recalculated and precisely weighed according to the exact mass of acetophenone, were subsequently added and the flask was flushed with argon. A freshly prepared 0.05 M solution of potassium hydroxide in propan‐2‐ol saturated with argon (4 mL) was added under the stream of argon and the flask stoppered firmly. The reaction mixture was heated in an oil bath at 100 °C for indicated time, after which it was immediately cooled to room temperature. An aliquot of the reaction mixture (0.2 mL) was transferred to an NMR tube, diluted with CDCl_3_ (0.5 mL) and ^1^H NMR spectrum was recorded. The conversion was determined by comparing the integral intensities of signals of the internal standard, 1,3,5‐trimethoxybenzene (s, 6.09 ppm), and of the expected product, 1‐phenylethanol (q, 4.86 ppm). For each system, the experiments were performed in triplicate. If the results of the independent runs differed significantly (more than 10 %), two more experiments were performed to support the reliability of the data.

## Results and Discussion

### Synthesis

As mentioned above, Rh(III) and Ir(III) cyclopentadienyl half‐sandwich complexes represent a prospective structural type for the development of new anticancer metallodrugs and fine catalysts. Chlorido complexes of this type are usually prepared from dimeric [M(μ‐Cl)(η^5^‐Cp*)Cl]_2_ compounds (M=Rh or Ir), the syntheses of which were previously optimized to be carried out in microwave reactors at 140 °C in less than 5 min.[^11^, ^12^] The less studied half‐sandwich Rh(III) and Ir(III) bromido and iodido complexes[^15^, ^19^, ^21^, ^22^, ^23^] can also be prepared from the dimeric complexes [M(μ‐Br)(η^5^‐Cp*)Br]_2_ and [M(μ‐I)(η^5^‐Cp*)I]_2_,[^9^, ^17^] but their syntheses[^20^, ^24^, ^25^] have not yet been optimized.

In this work, dinuclear complexes [Rh(μ‐Br)(η^5^‐Cp*)Br]_2_ (**1**), [Rh(μ‐I)(η^5^‐Cp*)I]_2_ (**2**), [Ir(μ‐Br)(η^5^‐Cp*)Br]_2_ (**3**) and [Ir(μ‐I)(η^5^‐Cp*)I]_2_ (**4**) (Figure 1) were prepared using a microwave‐assisted synthesis, and the reactions were optimized and streamlined for maximum simplification. In the synthesis procedure of bromido and iodido complexes **1**–**4**, the temperature (*i. e*., 140 °C) and solvent (MeOH) were left the same as previously used by Tonnemann *et al*. for the microwave‐assisted synthesis of [Rh(μ‐Cl)(η^5^‐Cp*)Cl]_2_ and [Ir(μ‐Cl)(η^5^‐Cp*)Cl]_2_,^[11]^ and only the reaction time was optimized. Regarding the recently described syntheses of Ir complexes **3** and **4**, acetone was utilized as the solvent at a temperature of 120 °C with a microwave reaction duration of 40 min.^[20]^ Additionally, this procedure involves a labour‐intensive isolation of the resulting products involving meticulous drying of products, re‐dissolution, triple extraction and filtration. In our experience, this preparation is time‐consuming, low yielding and very difficult to reproduce.

Our new microwave‐assisted method is simpler and has been optimized for time efficiency in the preparation of Ir complexes **3** and **4**. Furthermore, we extended these syntheses to include the rhodium(III) bromido (**1**) and iodido (**2**) dimers as well. These compounds (**1**–**4**) serve as important starting materials with applications ranging from the preparation of biological[^15^, ^18^, ^19^, ^21^, ^22^, ^23^] or catalytically[^26^, ^27^] active complexes. Our optimized synthetic methods are advantageous as they significantly reduce reaction times and simplify the preparation process.

### Characterization

The pentamethylcyclopentadienyl (Cp*) dimers **1**–**4** (Figure 1) were isolated as microcrystalline solids, partially soluble in MeOH, acetone and benzene, well‐soluble in dichloromethane and chloroform, and insoluble in water, hexane and DEE. Compounds **1**–**4** were studied by elemental analysis (<0.4 % differences between the theoretical and experimental contents of C and H), FTIR spectroscopy (Supporting information, Figure S1), ESI+ mass spectrometry (Supporting information, Figure S2–S5), and ^1^H and ^13^C NMR spectroscopy (Supporting information, Figure S6–S9).

The composition of the dimeric complexes **1**–**4** was confirmed by ESI+ mass spectra, which showed the peaks of the [M_2_X_3_(Cp*)_2_]^+^ and [MX(Cp*)]^+^ fragments of complexes (M=Rh or Ir, X=Br or I; Supporting information, Figure S2–S5). These cations formed by dehalogenation of **1**–**4**, which was in the case of [MX(Cp*)]^+^ species connected with fragmentation to mononuclear species.

The presence of the Cp* ligand in the structure of the complexes was detected in the ^1^H and ^13^C NMR spectra of **1**–**4** dissolved in DMSO‐*d_6_* (Supporting information, Figure S6–S9). In the ^1^H NMR spectra, the methyl group of the pentamethylcyclopentadienyl protons appears as a singlet in the range of 1.71–1.95 ppm. These characteristic resonances shifted downfield as compared to the chlorido precursors [Rh(μ‐Cl)(η^5^‐Cp*)Cl]_2_ (δ=1.62 ppm) and [Ir(μ‐Cl)(η^5^‐Cp*)Cl]_2_ (δ=1.63 ppm). The ^13^C NMR spectra of **1**–**4** exhibit resonances for the methyl carbons of the pentamethylcyclopentadienyl group in the range of 8.7–10.5 ppm and for the ring carbons at 93.0–100.8 ppm. These chemical shifts found in the ^13^C NMR spectra for **1**–**4** are, similarly to their ^1^H NMR spectra, higher as compared to the starting complexes [Rh(μ‐Cl)(η^5^‐Cp*)Cl]_2_ (δ=98.7, 8.5 ppm) and [Ir(μ‐Cl)(η^5^‐Cp*)Cl]_2_ (δ=92.0, 8.2 ppm).

Although no signals indicating the presence of chlorido precursors [Rh(μ‐Cl)(η⁵‐Cp*)Cl]₂ (for **1** and **2**) and [Ir(μ‐Cl)(η⁵‐Cp*)Cl]₂ (for **3** and **4**) were detected in the ^1^H and ^13^C NMR spectra of **1**–**4**, the possible presence of trace amounts of chlorine was further assessed using EDX. The analysis was conducted for **1**–**4**, as well as for their corresponding chlorido precursors [Rh(μ‐Cl)(η⁵‐Cp*)Cl]₂ and [Ir(μ‐Cl)(η⁵‐Cp*)Cl]₂ for comparative purposes (see Supporting Information, Figures S10,11).

The EDX measurements clearly confirmed the presence of rhodium (e. g., 2.697 keV; for [Rh(μ‐Cl)(η^5^‐Cp*)Cl]_2_, **1** and **2**), iridium (e. g., 9.175 keV; for [Ir(μ‐Cl)(η^5^‐Cp*)Cl]_2_, **3** and **4**), chlorine (e. g., 2.622 keV; for [Rh(μ‐Cl)(η^5^‐Cp*)Cl]_2_ and [Ir(μ‐Cl)(η^5^‐Cp*)Cl]_2_), bromine (e. g., 1.480 keV; for **1** and **3**) and iodine (e. g., 3.938 keV; for **2** and **4**). On the other hand, no signals corresponding to chlorine were detected in the EDX spectra of **1**–**4**, confirming the absence of Cl in these bromido and iodido dimers.

The FT‐IR spectra of complexes **1**–**4** showed the peaks of the characteristic vibrations of the Cp* ligand (Supporting information, Figure S1). For example, the peaks of the characteristic stretching ν(C−H)_aliphatic_ vibrations of the coordinated Cp* moiety were centred at 2900–2984 cm^−1^. The C−C stretching vibrations can be associated with the peaks observed in the range 1367–1473 cm^−1^.

### In Vitro Cytotoxicity

**Figure 2:**
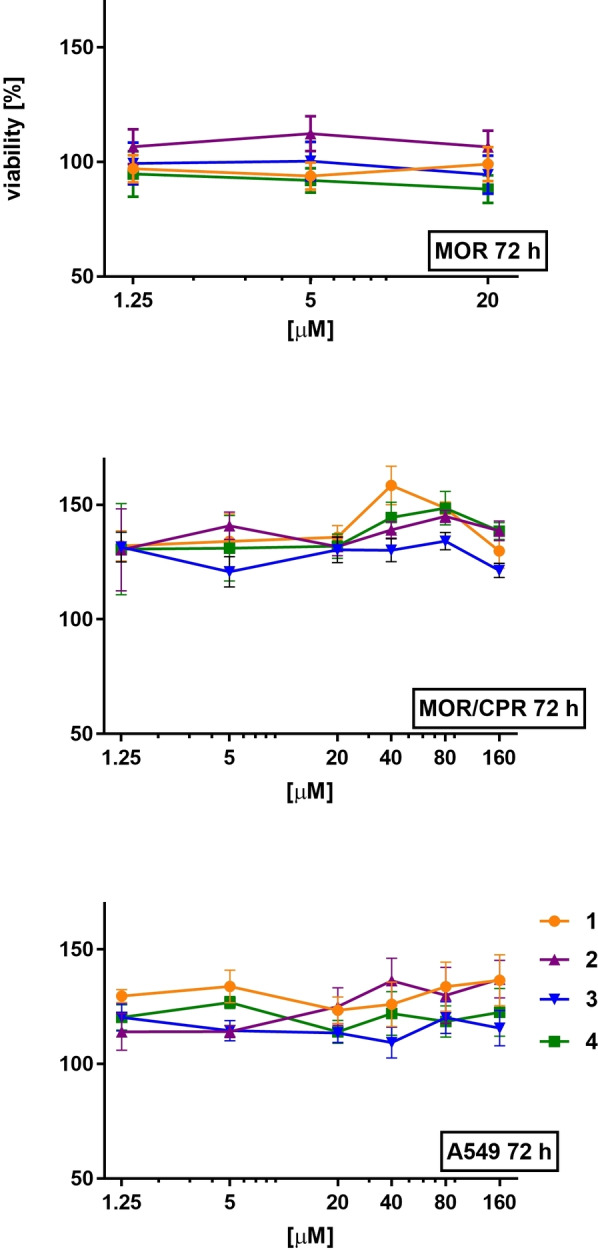
The effect of dimeric compounds **1**–**4** on the relative cell viability. Data are presented as mean ± standard error of mean (SEM). Cisplatin‐sensitive (MOR, A549) and ‐resistant (MOR/CPR) resistant human lung cancer cell lines were used for the screening.

As it is mentioned in the previous section, complexes **1**–**4** can be, for example, utilized as the starting material for the synthesis of biologically active Rh and Ir bromido and iodido complexes.[^15^, ^19^, ^21^, ^22^, ^23^] For a more objective evaluation of the biological activity of complexes created from such precursors, it is often useful to compare them with the starting compounds (such as **1**–**4**). Therefore, we performed cytotoxicity screening of the complexes studied here (**1**–**4**) on selected lung cancer cell lines (A549, MOR, and MOR/CPR; Figure 2). Synthetic precursors (*i. e*., HCp*, RhCl_3_⋅*x*H_2_O and IrCl_3_⋅*x*H_2_O) did not show any cytotoxic effect on A549 cells after 72 h incubation (data not shown).

Although cytotoxic effect of the starting dinuclear Rh(III) and Ir(III) chlorido complexes is well characterized,^[28]^ none of the tested bromido and iodido analogues **1**–**4** have been studied for their antiproliferative effect to date. We observed that **1**–**4** had no negative effect on the viability of the A549 and MOR/CRP cell lines up to 160 μM. Slight decrease in the viability of MOR cells (84 % viability) treated with 20 μM Ir iodido complex **4** was detected. Worth to note that even **4** is not possible to name as cytotoxic, because as cytotoxic molecules should be labelled only that with IC_50_ <10 μM, as was accepted by NIH to initial screen in the NCI60 program.^[29]^

Higher antiproliferative activity of Ir complex **4** than observed for its Rh analogue (**3**) correlates with the results formerly reported for Ir and Rh chlorido dimers, because complex [Ir(μ‐Cl)(η^5^‐Cp*)Cl]_2_ was more potent (IC_50_=47 μM) than [Rh(μ‐Cl)(η^5^‐Cp*)Cl]_2_ (IC_50_=81 μM); data given for 96 h incubation with A549 cells.^[28]^ Worth to note that cytotoxic effect is detectable only for long incubation time and high concentrations of tested complexes. With regard to the use of compounds **1**–**4** for the synthesis of biologically active half‐sandwich cyclopentadienyl Rh(III) and Ir(III) compounds, it can be stated that their cytotoxicity is negligible.

### Catalytic Transfer Hydrogenation of Acetophenone

As it was pointed out above, dimeric complexes of the general formula [M(μ‐X)(η^5^‐Cp*)X]_2_, where M=Rh, Ir and X=Cl, Br, I, are utilised as precatalysts in various reactions for which hydrogen transfer or hydrogen borrowing mechanism is usually proposed.[^30^, ^31^, ^32^, ^33^, ^34^, ^35^] For these applications, chlorido and iodido derivatives of this type are predominantly employed, whilst the bromido derivatives are used only scarcely. Surprisingly, only few reports compared the catalytic activities of the whole series of complexes.^[36]^ Having all the mentioned halogenido rhodium and iridium dimeric complexes in our hands, we decided to evaluate their catalytic performance to see what effect the central metal ion and the halogenido ligand have on the precatalyst activity.

**Figure 3:**
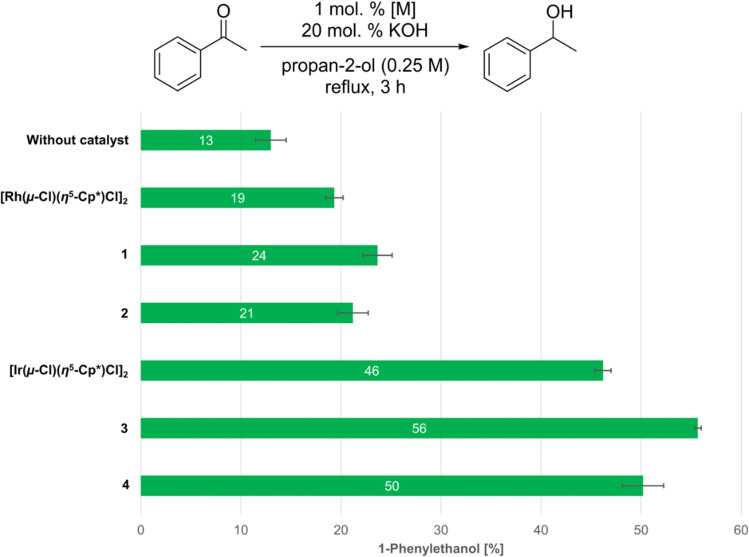
Catalytic activity of [M(μ‐X)(η^5^‐Cp*)X]_2_ complexes in transfer hydrogenation of acetophenone. Conversions were determined by integration of the NMR spectra of the reaction mixture using 1,3,5‐trimethoxybenzene as an internal standard and are presented as an average of at least 3 independent runs. Error bars represent standard error of the mean (SEM).

Transfer hydrogenation of acetophenone using propan‐2‐ol as the hydrogen source was selected as a model reaction. This reaction became almost standard for testing the transfer hydrogenation activity of platinum‐group metal complexes, mainly due to its simplicity and easy monitoring by NMR or GC techniques.[^37^, ^38^] For **1**–**4**, the reaction conditions were, with small modifications, adapted from reference.^[38]^ Specifically, 0.25 M acetophenone solution in propan‐2‐ol was heated to reflux in the presence of 20 mol. % of potassium hydroxide and 1 mol. % (metal basis) of respective precatalyst for 3 h, when the reaction mixture was analysed by NMR spectroscopy using 1,3,5‐trimethoxybenzene as an internal quantitative standard. The results are summarised in Figure 3.

In all cases, acetophenone was reduced selectively to 1‐phenylethanol with simultaneous oxidation of propan‐2‐ol to acetone and no other products were recognised in the NMR spectra of the reaction mixtures. The reaction proceeded slowly (13 % conversion) even without the addition of any catalyst which agrees with previously published reports.^[38]^ The conversion to 1‐phenylethanol increased only slightly upon addition of the rhodium‐based complexes as catalysts, being in the range of 19–24 %. Interestingly, the apparent order of activity according to the halogenido ligand was Br > I > Cl for the tested Rh compounds. On the other hand, moderate conversions ranging from 46 to 56 % were achieved using iridium‐based complexes. The same halogenido ligand‐activity relationship as for rhodium complexes was found (*i. e*., Br > I > Cl), with Ir(III) bromido complex **3** being the best performing precatalyst from the whole series. This order is different than found in the 1970s when hydrogenation activities of [M(μ‐X)(η^5^‐Cp*)X]_2_ complexes using hydrogen gas as hydrogen source was tested (Cl > Br > I).^[39]^ Nevertheless, even this review summarizing the pioneer investigations performed by Maitlis and co‐workers reported Ir dimers as outperforming their Rh congeners.

The observed structure‐activity relationship is somewhat surprising and probably arises from a delicate interplay between factors such as the ease of metal‐halogen bond cleavage, the ability of remaining halogenido ligands to stabilise catalytically relevant intermediates, and the influence of the halogenido ligands on the electron density of the metal centre, with no one of these factors prevailing. The interpretation could be that in the case of bromido complexes, the advantageous and disadvantageous effects average and, therefore, the bromido derivatives are the most active ones. Despite the unclear mechanism, the catalytic activity of the dimers studied here (especially **3** and **4**) is very important in terms of discussing the similar activity of Rh and Ir complexes prepared from the dinuclear synthons discussed here.

## Conclusions

In this work, the synthesis of dinuclear complexes [Rh(μ‐Br)(η^5^‐Cp*)Br]_2_ (**1**), [Rh(μ‐I)(η^5^‐Cp*)I]_2_ (**2**), [Ir(μ‐Br)(η^5^‐Cp*)Br]_2_ (**3**) and [Ir(μ‐I)(η^5^‐Cp*)I]_2_ (**4**), which represent the intermediates used in the syntheses of half‐sandwich bromido and iodido complexes of these metals, was successfully optimized. The synthesis is carried out in a microwave reaction system at 140 °C, is reproducible and provides high yields of compounds that are stable and well storable. Model studies of *in vitro* antiproliferative activity have shown that these compounds are not cytotoxic to cisplatin‐sensitive (A549, MOR) and ‐resistant (MOR/CPR) human lung cancer cell lines. Iridium complexes **3** and **4** exhibited moderate catalytic activity in a model transfer hydrogenation of acetophenone in propan‐2‐ol, whereas the activity of the rhodium analogues **1** and **2** was negligible. The bromido complexes showed higher hydrogenation activity within the halogenido series, outperforming their iodido and chlorido analogues.

## Notes

The authors declare no competing financial interest. Data are available on request from the corresponding author.

## Author Contributions

The manuscript was written through contributions of all authors. All authors have given approval to the final version of the manuscript.

## Conflict of Interests

The authors declare no conflict of interest.

## Supporting information

As a service to our authors and readers, this journal provides supporting information supplied by the authors. Such materials are peer reviewed and may be re‐organized for online delivery, but are not copy‐edited or typeset. Technical support issues arising from supporting information (other than missing files) should be addressed to the authors.

Supporting Information

## Data Availability

The data that support the findings of this study are available from the corresponding author upon reasonable request.
