# Supplementary material for: Improved Synthesis of Dinuclear [M(μ‐X)(η5‐Cp*)X]2 Precursors for Half‐Sandwich Complexes (M=Rh or Ir; X=Br or I)
Source: ChemistryOpen. 2025 Mar 3;14(9):e202500026. doi: 10.1002/open.202500026 (PMC12409828; doi:10.1002/open.202500026)
Supplement: Supplementary file 1 — Supporting Information [file OPEN-14-e202500026-s001.pdf]

ChemistryOpen

Supporting Information

Improved Synthesis of Dinuclear [M(μ-X)(η^5-Cp*)X]$_2$ Precursors for Half-Sandwich Complexes (M = Rh or Ir; X = Br or I)

Kamila Petrželová, Ondřej Bárta, Renata Héžová, Alžběta Andrýsková, Jan Hošek, and Pavel Štarha*

Supporting information

for

Improved Synthesis of Dinuclear $[M(\mu\text{-}X)(\eta^5\text{-}Cp^*)X]_2$ Precursors for Half-Sandwich Complexes (M = Rh or Ir; X = Br or I)

Kamila Petrželová [a], Ondřej Bárta [a], Renata Héžová [b], Alžběta Andrýsková [c], Jan Hošek [b], Pavel Štarha [a,*]

[a] *Department of Inorganic Chemistry, Faculty of Science, Palacký University Olomouc, 17. listopadu 1192/12, 77146 Olomouc, Czech Republic*

[b] *Department of Pharmacology and Toxicology, Veterinary Research Institute, Hudcova 296/70, 62100 Brno, Czech Republic*

[c] *Department of Experimental Physics, Faculty of Science, Palacký University Olomouc, 17. listopadu 1192/12, 77146 Olomouc, Czech Republic*

* Corresponding Author:

E-mail: pavel.starha@upol.cz; phone +420 585 634 348

Table of Contents

Figure S1 - FT-IR spectra of complexes **1–4** S3

Figure S2–S5 - ESI+ mass spectra of complexes **1–4** S4

Figure S6–S9 - ^{1}H and ^{13}C NMR spectra of complexes **1–4** S8

Figure S10,11 - EDX spectra of complexes **1–4** S12

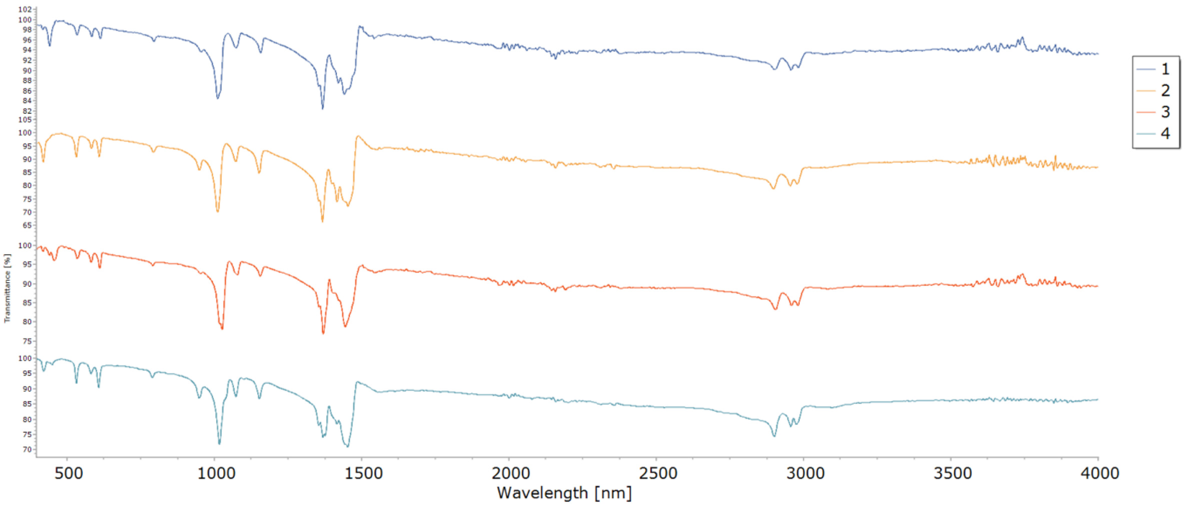

Figure S1. FT-IR spectra of complexes **1–4.**

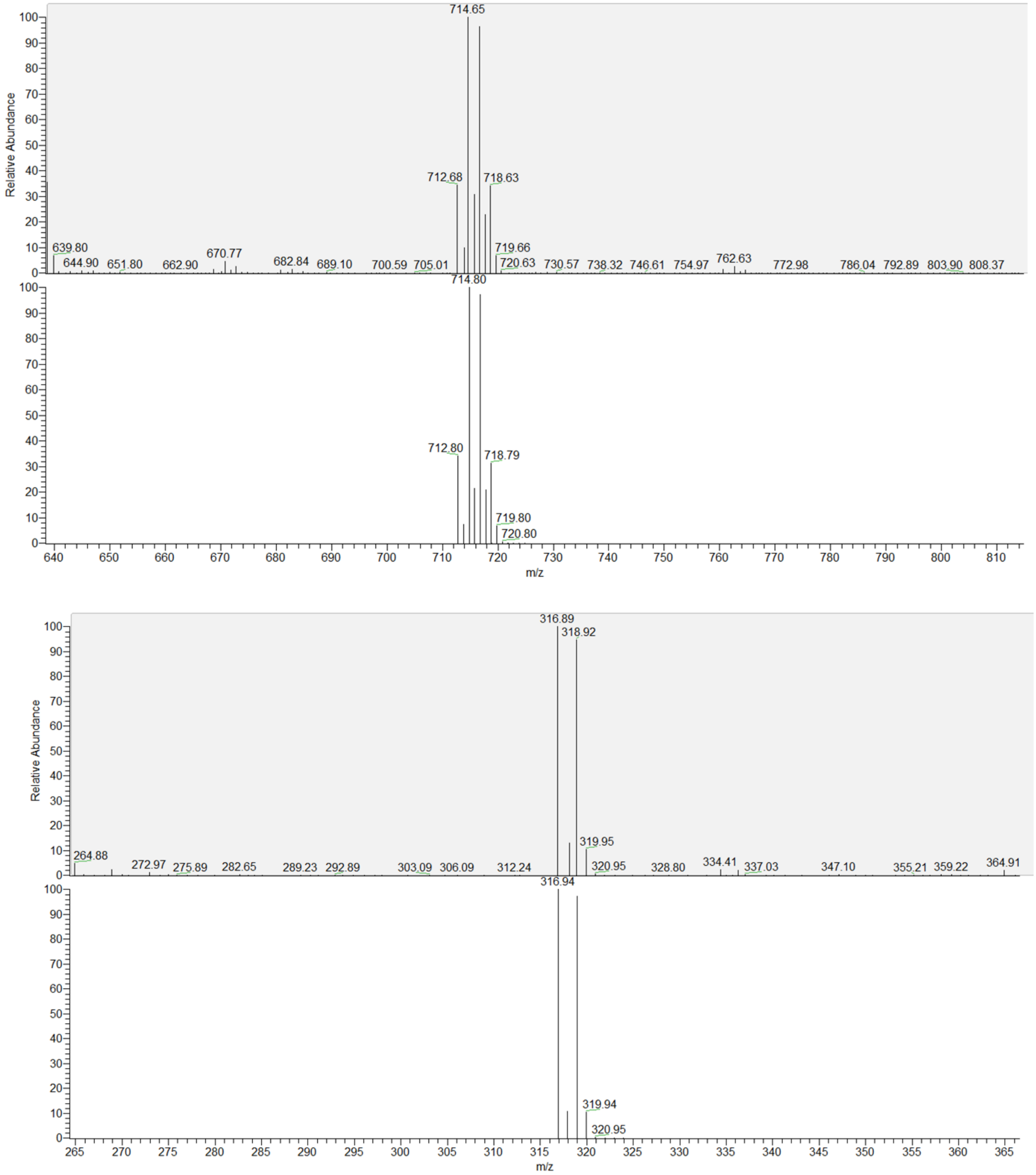

Figure S2. ESI+ mass spectra of complex **1**, given with a comparison of the experimental (*grey*) and theoretical (*white*) isotopic pattern calculated for the $[Rh_2Br_3(Cp^*)_2)]^+$ (*top*) and $[RhBr(Cp^*)]^+$ (*bottom*) species. ESI+ = positive electrospray ionization mode. Complex **1** was dissolved in MeOH.

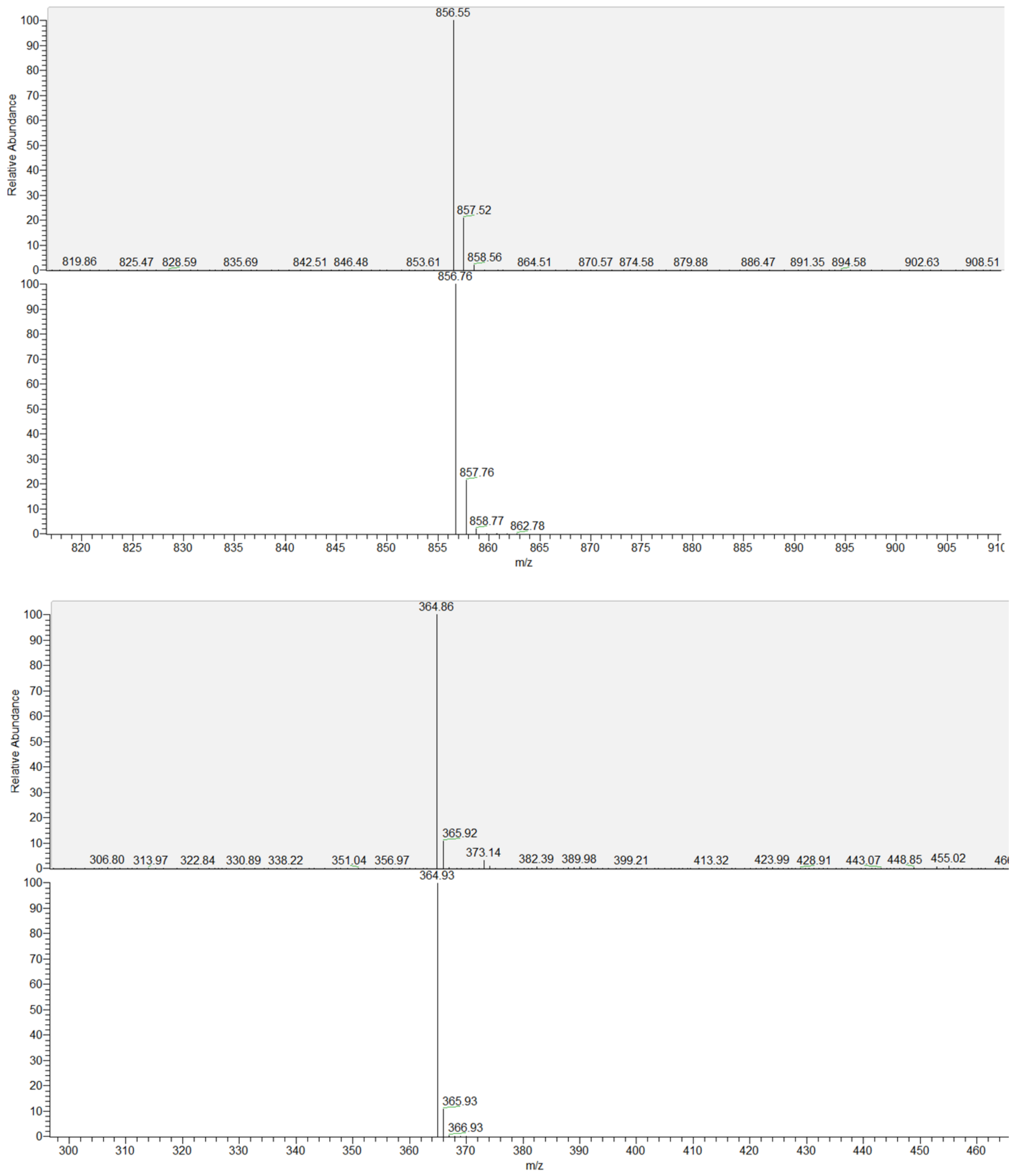

Figure S3. ESI+ mass spectra of complex **2**, given with a comparison of the experimental (*grey*) and theoretical (*white*) isotopic pattern calculated for the $[Rh_2I_3(Cp^*)_2]^+$ (*top*) and $[RhI(Cp^*)]^+$ (*bottom*) species. ESI+ = positive electrospray ionization mode. Complex **2** was dissolved in MeOH.

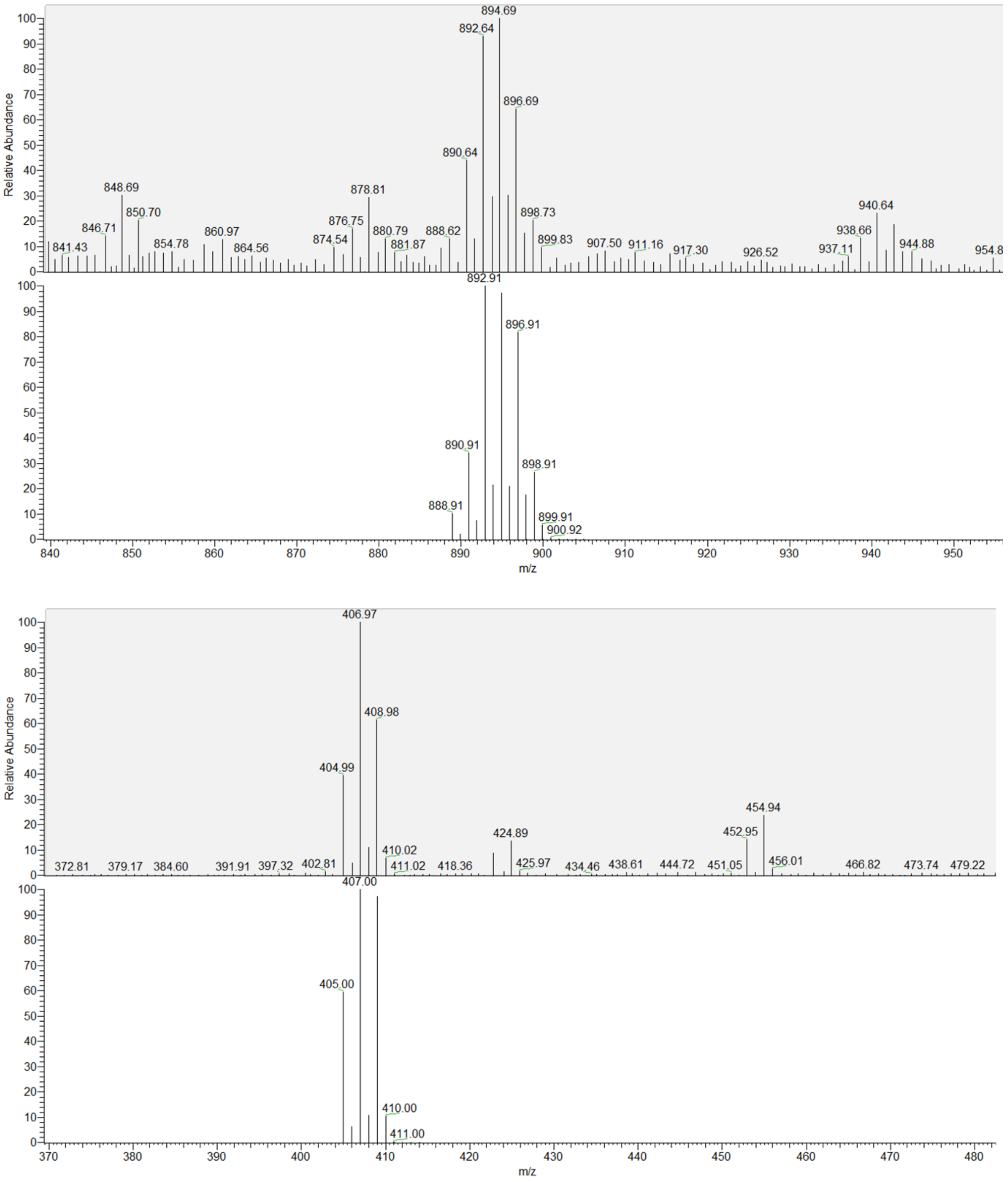

Figure S4. ESI+ mass spectra of complex **3**, given with a comparison of the experimental (*grey*) and theoretical (*white*) isotopic pattern calculated for the $[Ir_2Br_3(Cp^*)_2)]^+$ (*top*) and $[IrBr(Cp^*)]^+$ (*bottom*) species. ESI+ = positive electrospray ionization mode. Complex **3** was dissolved in MeOH.

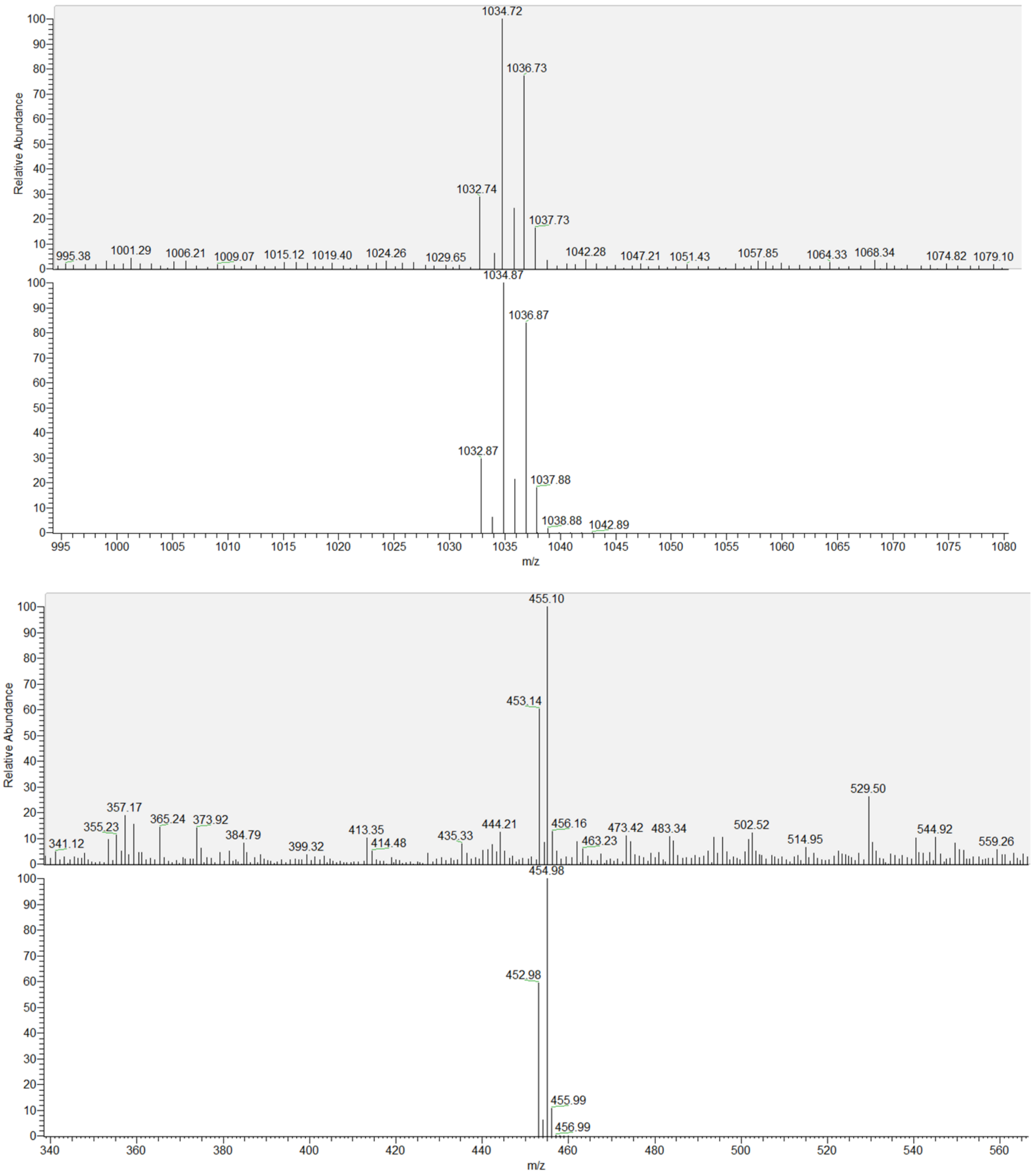

Figure S5. ESI+ mass spectra of complex **4**, given with a comparison of the experimental (*grey*) and theoretical (*white*) isotopic pattern calculated for the $[Ir_2I_3(Cp^*)_2)]^+$ (*top*) and $[IrI(Cp^*)]^+$ (*bottom*) species. ESI+ = positive electrospray ionization mode. Complex **4** was dissolved in MeOH.

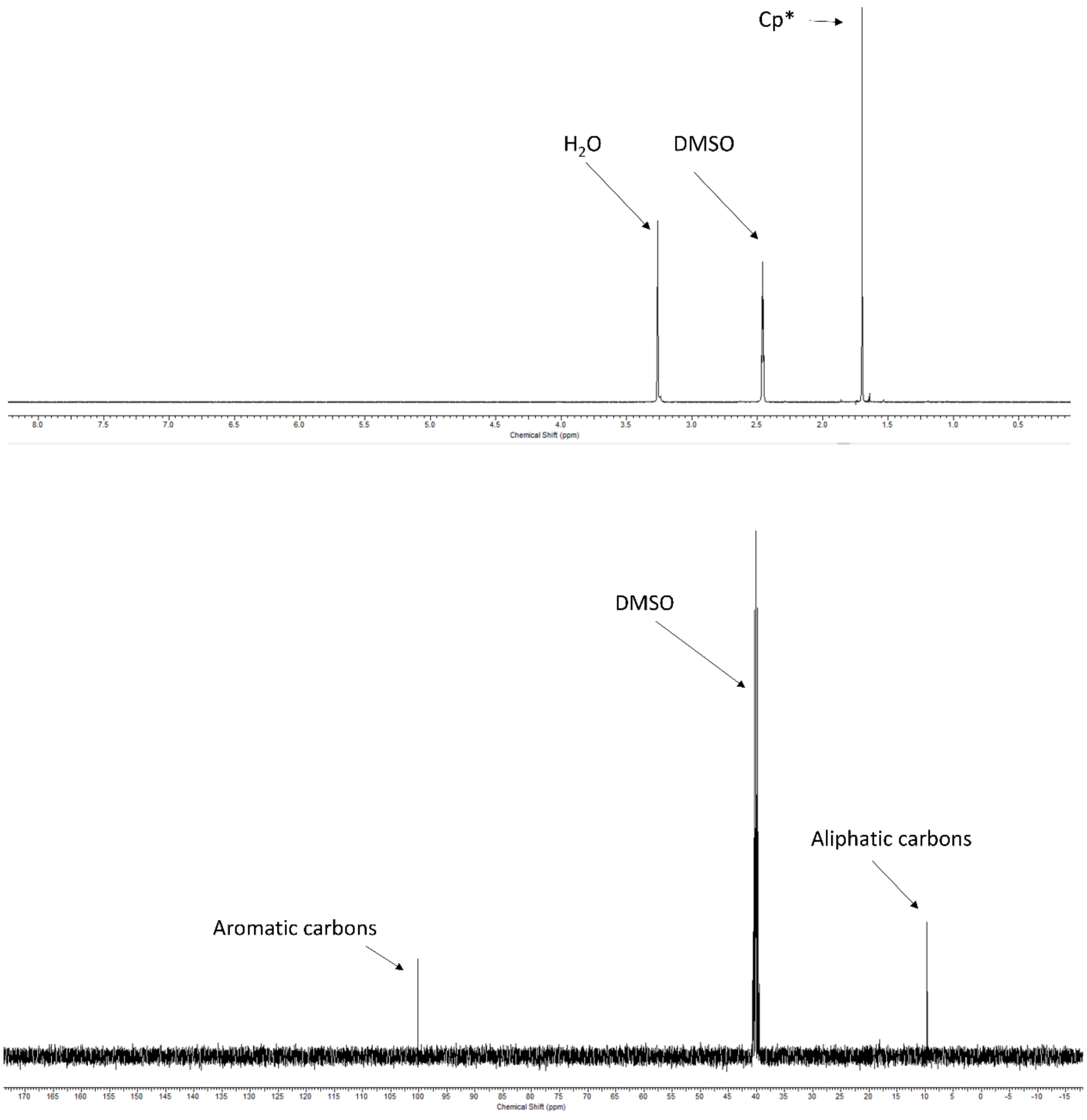

Figure S6. ^{1}H (*top*) and ^{13}C (*bottom*) NMR spectra of complex **1** in DMSO-*d*$_6$.

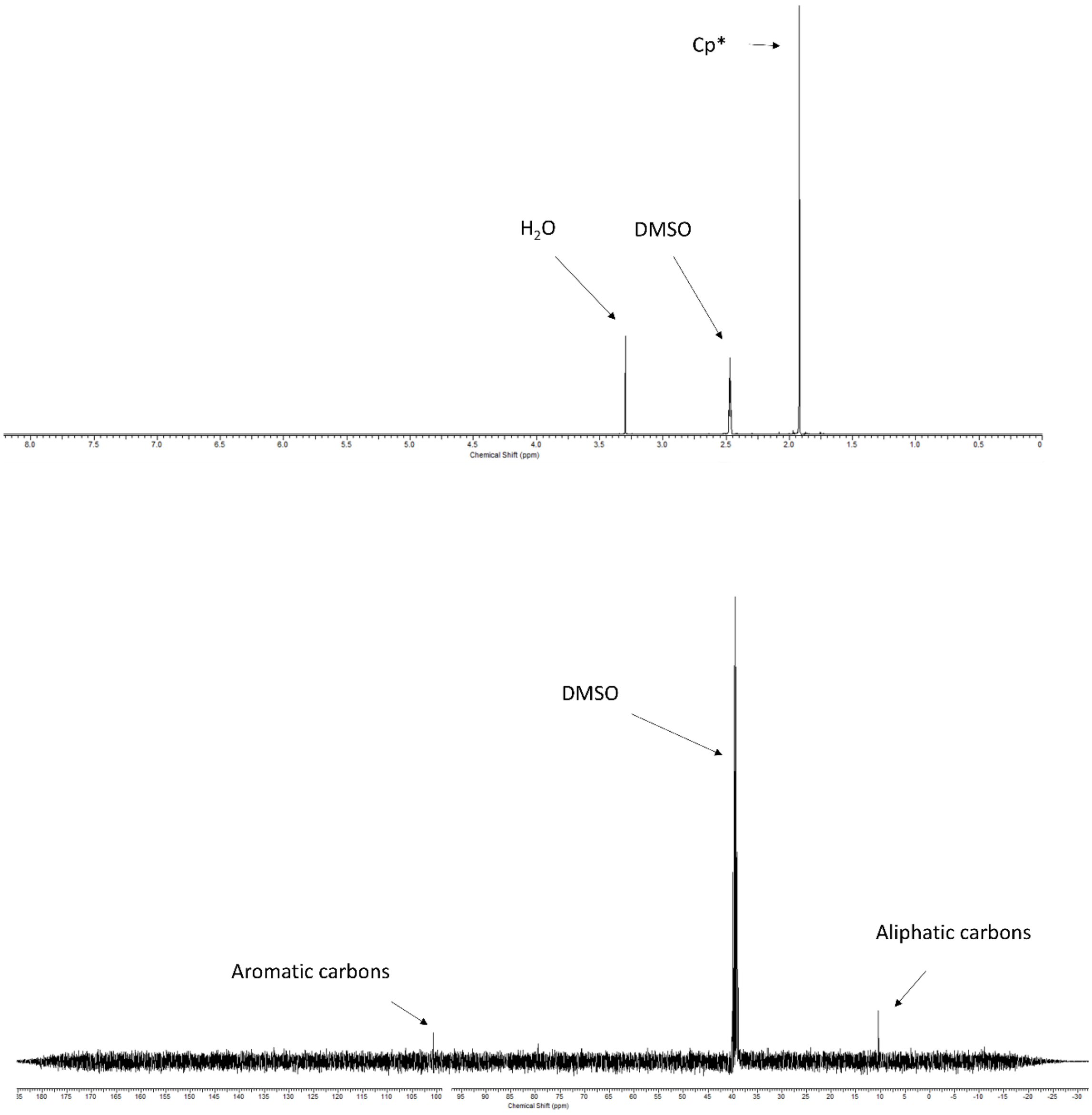

Figure S7. ^{1}H (*top*) and ^{13}C (*bottom*) NMR spectra of complex **2** in DMSO-d_6.

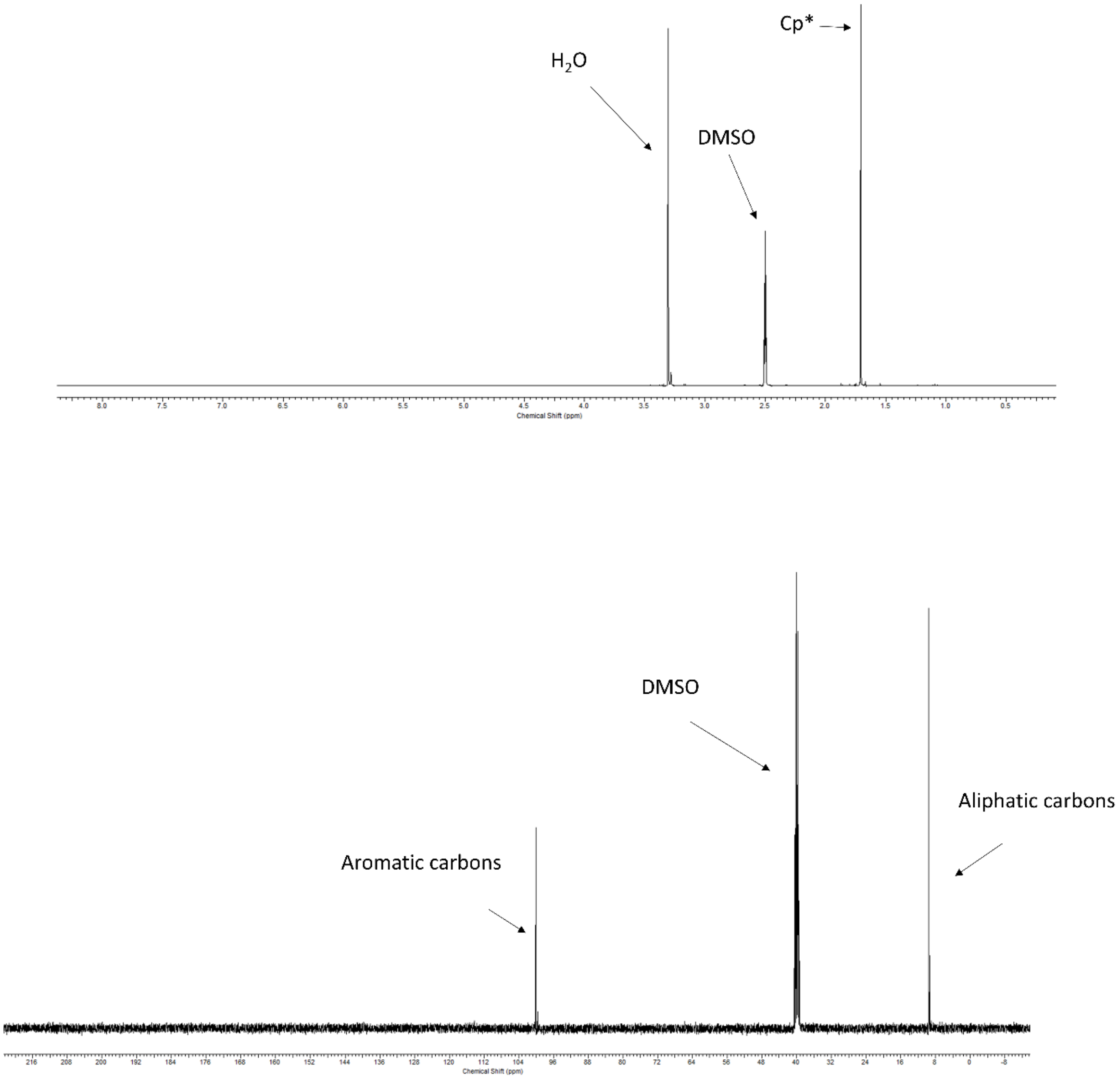

Figure S8. ^{1}H (*top*) and ^{13}C (*bottom*) NMR spectra of complex **3** in DMSO-*d*$_6$.

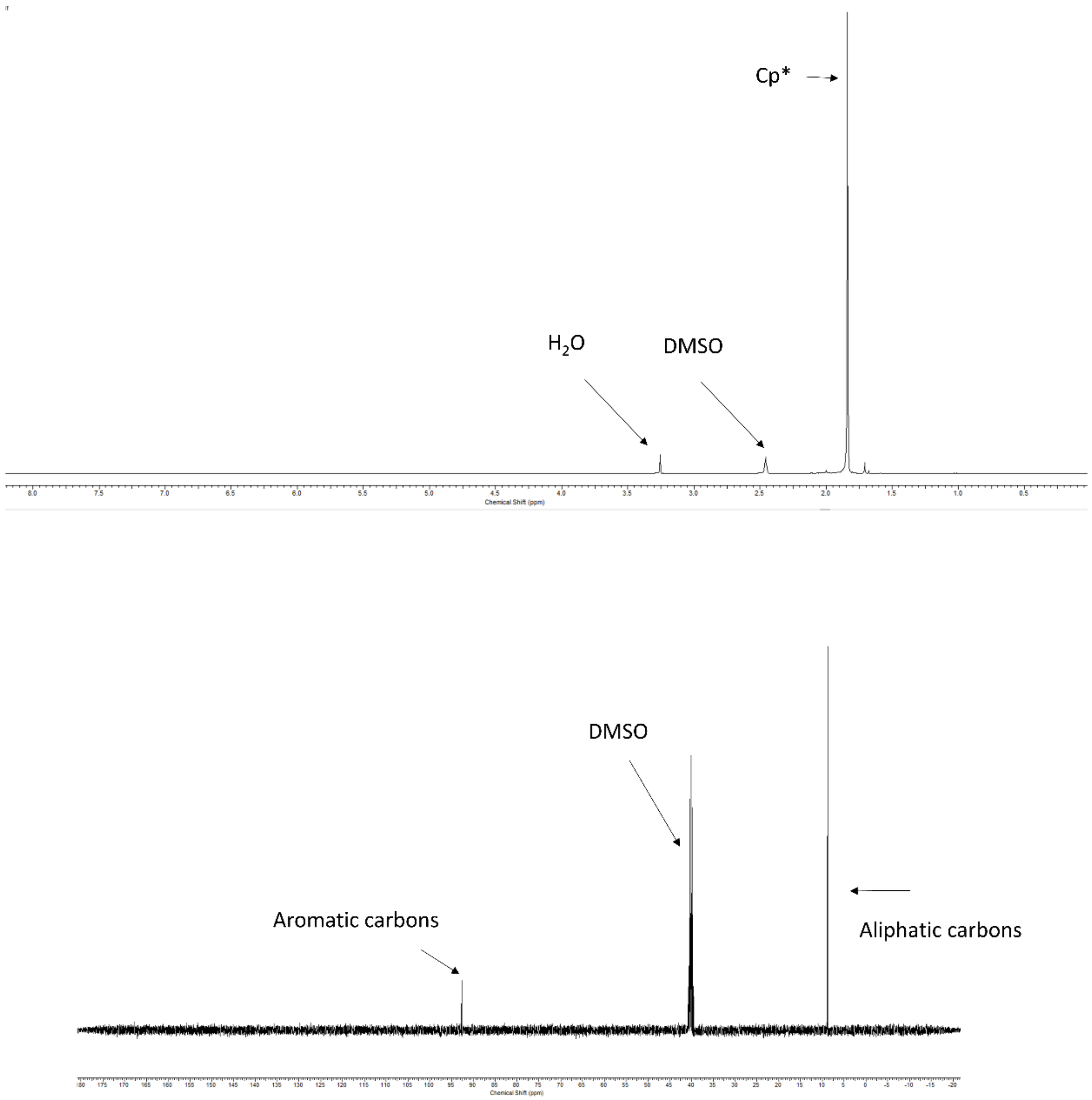

Figure S9. ^{1}H (*top*) and ^{13}C (*bottom*) NMR spectra of complex **4** in DMSO-*d*$_6$.

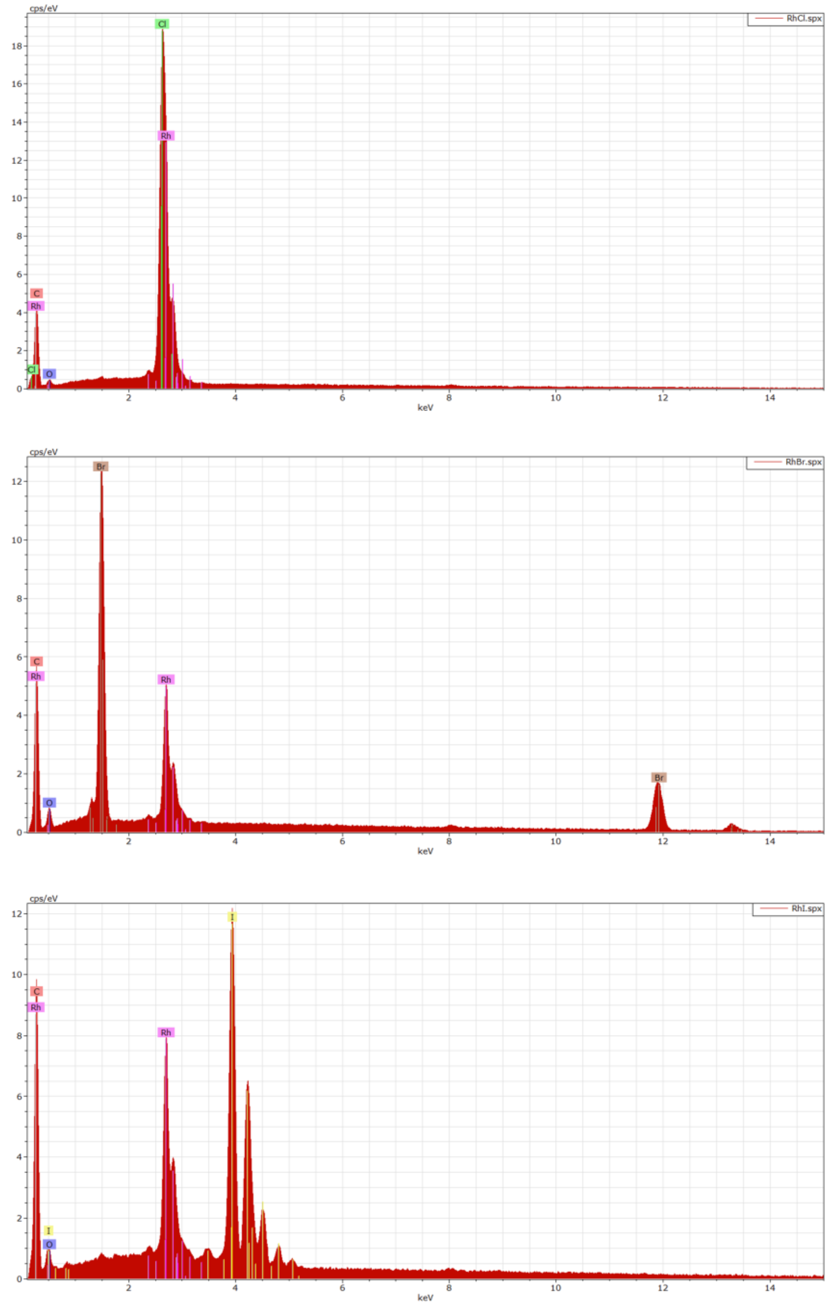

Figure S10. EDX spectra of the rhodium(III) compounds [Rh(μ-Cl)(η^5-Cp*)Cl]$_2$ (*top*), [Rh(μ-Br)(η^5-Cp*)Br]$_2$ (**1**; *middle*) and [Rh(μ-I)(η^5-Cp*)I]$_2$ (**2**; *bottom*), with the assigned peaks of rhodium (*e.g.*, 2.697 keV), chlorine (*e.g.*, 2.622 keV), bromine (*e.g.*, 1.480 keV) and iodine (*e.g.*, 3.938 keV).

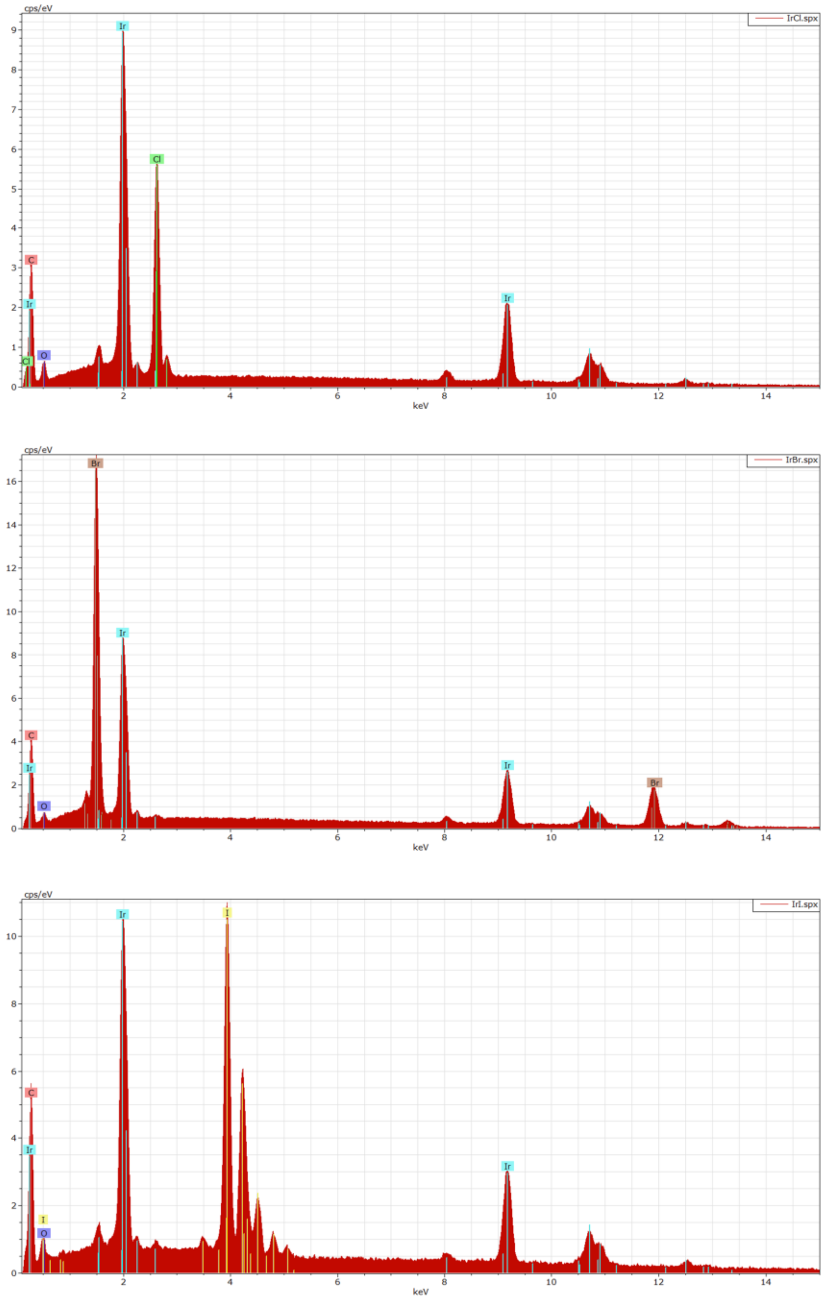

Figure S11. EDX spectra of the iridium(III) compounds $[Ir(\mu\text{-}Cl)(\eta^5\text{-}Cp^*)Cl]_2$ (*top*), $[Ir(\mu\text{-}Br)(\eta^5\text{-}Cp^*)Br]_2$ (**3**; *middle*) and $[Ir(\mu\text{-}I)(\eta^5\text{-}Cp^*)I]_2$ (**4**; *bottom*), with the assigned peaks of iridium (*e.g.*, 9.175 keV), chlorine (*e.g.*, 2.622 keV), bromine (*e.g.*, 1.480 keV) and iodine (*e.g.*, 3.938 keV).
